# 6-Chloro-3-[5-(3-meth­oxy-8-methyl-4-quinol­yl)-1-phenyl-4,5-dihydro-1*H*-pyrazol-3-yl]-2-methyl-4-phenyl­quinoline

**DOI:** 10.1107/S1600536809040252

**Published:** 2009-10-07

**Authors:** Hoong-Kun Fun, Chin Sing Yeap, S. Sarveswari, V. Vijayakumar, R. Prasath

**Affiliations:** a X-ray Crystallography Unit, School of Physics, Universiti Sains Malaysia, 11800 USM, Penang, Malaysia; b Organic Chemistry Division, School of Sciences, VIT University, Vellore 632 014, India

## Abstract

In the title compound, C_36_H_29_ClN_4_O, the dihydro­pyrazole ring adopts an envelope conformation. The two quinoline ring systems (r.m.s. deviations = 0.029 and 0.018 Å) are oriented at a dihedral angle of 71.43 (4)°. One of the quinoline rings makes a dihedral angle of 65.40 (7)° with the phenyl substituent. In the crystal, mol­ecules are linked into chains along the *b* axis by inter­molecular C—H⋯N hydrogen bonds. In addition, C—H⋯π and π–π [centroid–centroid distance = 3.7325 (8) Å] inter­actions are observed.

## Related literature

For general background to quinoline and its derivatives, see: Morimoto *et al.* (1991 ▶); Michael (1997 ▶); Markees *et al.* (1970 ▶); Campbell *et al.* (1988 ▶). For applications of quinolines, see: Maguire *et al.* (1994 ▶); Kalluraya & Sreenivasa (1998 ▶); Roma *et al.* (2000 ▶); Chen *et al.* (2001 ▶); Skraup (1880 ▶). For the synthesis of quinoline derivatives, see: Katritzky & Arend (1998 ▶); Jiang & Si (2002 ▶). For ring conformations, see: Cremer & Pople (1975 ▶). For the stability of the temperature controller used for the data collection, see: Cosier & Glazer (1986 ▶).
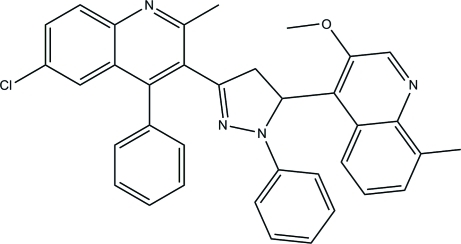


## Experimental

### 

#### Crystal data

C_36_H_29_ClN_4_O
*M* *_r_* = 569.08
Monoclinic, 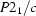

*a* = 14.1209 (3) Å
*b* = 20.2273 (4) Å
*c* = 10.1892 (2) Å
β = 95.358 (1)°
*V* = 2897.6 (1) Å^3^
*Z* = 4
Mo *K*α radiation
μ = 0.17 mm^−1^
*T* = 100 K
0.65 × 0.45 × 0.22 mm

#### Data collection

Bruker SMART APEXII CCD area-detector diffractometer
Absorption correction: multi-scan (**SADABS**; Bruker, 2005 ▶) *T*_min_ = 0.899, *T*_max_ = 0.964
57308 measured reflections
10615 independent reflections
8632 reflections with *I* > 2σ(*I*)
*R*_int_ = 0.032

#### Refinement

*R*[*F* ^2^ > 2σ(*F* ^2^)] = 0.056
*wR*(*F* ^2^) = 0.135
*S* = 1.08
10615 reflections
495 parameters
All H-atom parameters refined
Δρ_max_ = 0.52 e Å^−3^
Δρ_min_ = −0.38 e Å^−3^

### 

Data collection: *APEX2* (Bruker, 2005 ▶); cell refinement: *SAINT* (Bruker, 2005 ▶); data reduction: *SAINT*; program(s) used to solve structure: *SHELXTL* (Sheldrick, 2008 ▶); program(s) used to refine structure: *SHELXTL*; molecular graphics: *SHELXTL*; software used to prepare material for publication: *SHELXTL* and *PLATON* (Spek, 2009 ▶).

## Supplementary Material

Crystal structure: contains datablocks global, I. DOI: 10.1107/S1600536809040252/ci2931sup1.cif

Structure factors: contains datablocks I. DOI: 10.1107/S1600536809040252/ci2931Isup2.hkl

Additional supplementary materials:  crystallographic information; 3D view; checkCIF report

## Figures and Tables

**Table 1:** Hydrogen-bond geometry (Å, °)

*D*—H⋯*A*	*D*—H	H⋯*A*	*D*⋯*A*	*D*—H⋯*A*
C13—H13⋯N1^i^	0.95 (2)	2.56 (2)	3.410 (2)	149 (2)
C14—H14⋯*Cg*1	1.00 (2)	2.92 (2)	3.685 (2)	134 (2)
C17—H17*A*⋯*Cg*2^ii^	0.97 (2)	2.55 (2)	3.5018 (14)	169 (1)
C25—H25⋯*Cg*3^iii^	0.96 (2)	2.79 (2)	3.7359 (17)	169 (2)

Symmetry codes: (i) 
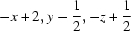
; (ii) 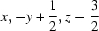; (iii) 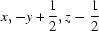. *Cg*1, *Cg*2 and *Cg*3 are centroids of the C21–C26, N1/C1/C6–C9 and C28–C33 benzene rings, respectively.

**Fig. 1.**
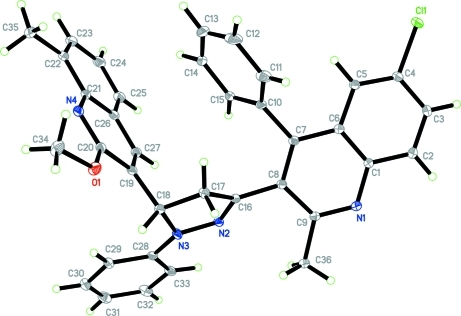
The molecular structure of the title compound, with atom labels and 50% probability ellipsoids for non-H atoms.

**Fig. 2.**
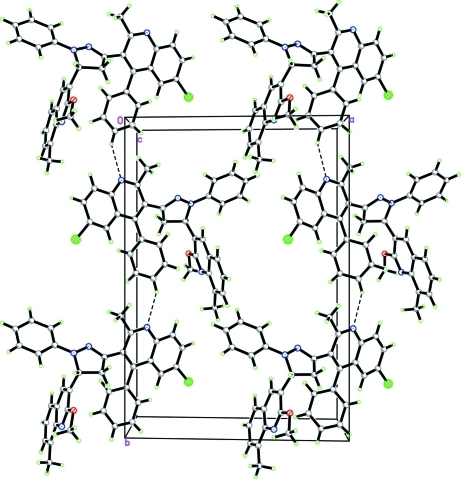
The crystal packing of title compound, viewed down c axis, showing molecules linked down the b axis. Hydrogen bonds are shown as dashed lines.

## supplementary crystallographic information

### Comment

Quinoline and its derivatives are very important compounds because of their wide occurrence in natural products (Morimoto *et al.*, 1991; Michael, 1997) and biologically active compounds (Markees *et al.*, 1970; Campbell *et al.*, 1988). A large variety of quinolines have interesting physiological activities and found attractive applications as pharmaceuticals, agrochemicals and as synthetic building blocks (Maguire *et al.*, 1994; Kalluraya & Sreenivasa, 1998; Roma *et al.*, 2000; Chen *et al.*, 2001; Skraup, 1880). Many synthetic methods such as Skraup, Doebner-Von Miller, Friedländer and Combes reactions have been developed for the preparation of quinolines, but due to their great importance, the synthesis of new derivatives of quinoline remains an active research area (Katritzky & Arend, 1998; Jiang & Si, 2002).

The title compound consists of two phenyl rings, two quinoline rings and a 4,5-dihydropyrazole ring (Fig. 1). The 4,5-dihydropyrazole ring (N2/N3/C16–C18) adopts an envelope conformation with C18 as the flap atom, and with puckering amplitude Q = 0.2121 (14) Å and φ = 256.8 (3)° (Cremer & Pople, 1975). The N1/C1–C9 quinoline ring is planar with a maximum deviation of 0.046 (1) Å for atom C8 and in the N4/C19–C27 ring atom C19 deviates a maximum of 0.034 (1) Å. The two quinoline rings (N1/C1–C9 and N4/C19–C27) are oriented at angle of 71.43 (4)°. The C10–C15 benzene ring makes a dihedral angle of 65.40 (7)° with the N1/C1–C9 quinoline ring.

In the crystal structure, molecules are linked into chains along the *b* axis (Fig. 2) by intermolecular C13—H13···N1 hydrogen bonds (Table 1). In addition, the structure is stabilized by the C—H···π (Table 1) and π···π interactions [*Cg*1···*Cg*1^iv^ = 3.7325 (8) Å; *Cg*1 is centroid of C21–C26 ring; (iv) 1 - *x*, 1 - *y*, -*z*].

### Experimental

A mixture of 1-(6-chloro-2-methyl-4-phenylquinolin-3-yl)-3- (2-chloroquinolin-2-yl)prop-2-en-1-one (0.468 g, 0.001 *M*) and phenyl hydrazine in (0.756 g, 0.007 *M*) in distilled methanol was refluxed for 8 h and the resulting mixture was concentrated to remove the methanol and then poured onto ice and neutralized with dilute HCl. The resultant solid was filtered, dried and purified by column chromatography using 1:1 mixture of chloroform and petroleum ether. The resultant product was recrystallized from methanol (m.p. 456–458 K).

### Refinement

All H atoms were located in a difference Fourier map and refined freely [C–H = 0.914 (18)–1.000 (20) Å].

### Crystal data

**Table:**

C_36_H_29_ClN_4_O	*F*(000) = 1192
*M**_r_* = 569.08	*D*_x_ = 1.305 Mg m^−^^3^
Monoclinic, *P*2_1_/*c*	Mo *K*α radiation, λ = 0.71073 Å
Hall symbol: -P 2ybc	Cell parameters from 9113 reflections
*a* = 14.1209 (3) Å	θ = 2.3–32.7°
*b* = 20.2273 (4) Å	µ = 0.17 mm^−^^1^
*c* = 10.1892 (2) Å	*T* = 100 K
β = 95.358 (1)°	Block, orange
*V* = 2897.6 (1) Å^3^	0.65 × 0.45 × 0.22 mm
*Z* = 4	

### Data collection

**Table:**

Bruker SMART APEXII CCD area-detector diffractometer	10615 independent reflections
Radiation source: fine-focus sealed tube	8632 reflections with *I* > 2σ(*I*)
graphite	*R*_int_ = 0.032
φ and ω scans	θ_max_ = 32.7°, θ_min_ = 2.0°
Absorption correction: multi-scan (*SADABS*; Bruker, 2005)	*h* = −15→21
*T*_min_ = 0.899, *T*_max_ = 0.964	*k* = −30→27
57308 measured reflections	*l* = −15→15

### Refinement

**Table:**

Refinement on *F*^2^	Primary atom site location: structure-invariant direct methods
Least-squares matrix: full	Secondary atom site location: difference Fourier map
*R*[*F*^2^ > 2σ(*F*^2^)] = 0.056	Hydrogen site location: inferred from neighbouring sites
*wR*(*F*^2^) = 0.135	All H-atom parameters refined
*S* = 1.08	*w* = 1/[σ^2^(*F*_o_^2^) + (0.0511*P*)^2^ + 1.8405*P*] where *P* = (*F*_o_^2^ + 2*F*_c_^2^)/3
10615 reflections	(Δ/σ)_max_ = 0.001
495 parameters	Δρ_max_ = 0.52 e Å^−^^3^
0 restraints	Δρ_min_ = −0.38 e Å^−^^3^

### Special details

**Table:**

Experimental. The crystal was placed in the cold stream of an Oxford Cyrosystems Cobra open-flow nitrogen cryostat (Cosier & Glazer, 1986) operating at 100.0 (1) K.
Geometry. All e.s.d.'s (except the e.s.d. in the dihedral angle between two l.s. planes) are estimated using the full covariance matrix. The cell e.s.d.'s are taken into account individually in the estimation of e.s.d.'s in distances, angles and torsion angles; correlations between e.s.d.'s in cell parameters are only used when they are defined by crystal symmetry. An approximate (isotropic) treatment of cell e.s.d.'s is used for estimating e.s.d.'s involving l.s. planes.
Refinement. Refinement of *F*^2^ against ALL reflections. The weighted *R*-factor *wR* and goodness of fit *S* are based on *F*^2^, conventional *R*-factors *R* are based on *F*, with *F* set to zero for negative *F*^2^. The threshold expression of *F*^2^ > σ(*F*^2^) is used only for calculating *R*-factors(gt) *etc*. and is not relevant to the choice of reflections for refinement. *R*-factors based on *F*^2^ are statistically about twice as large as those based on *F*, and *R*- factors based on ALL data will be even larger.

### Fractional atomic coordinates and isotropic or equivalent isotropic displacement parameters (Å^2^)

**Table:**

	*x*	*y*	*z*	*U*_iso_*/*U*_eq_	
Cl1	1.25829 (2)	0.618905 (17)	0.58916 (3)	0.02412 (8)	
O1	0.74552 (8)	0.56821 (5)	−0.26011 (9)	0.0227 (2)	
N1	1.06317 (8)	0.82047 (6)	0.24624 (12)	0.0199 (2)	
N2	0.79102 (8)	0.76177 (5)	0.05659 (10)	0.0165 (2)	
N3	0.72868 (8)	0.74397 (5)	−0.05041 (11)	0.0184 (2)	
N4	0.67553 (8)	0.50533 (6)	−0.10704 (11)	0.0192 (2)	
C1	1.10707 (9)	0.77113 (6)	0.32078 (14)	0.0194 (2)	
C2	1.18398 (11)	0.78906 (8)	0.41257 (17)	0.0300 (3)	
C3	1.22949 (11)	0.74267 (8)	0.49378 (17)	0.0293 (3)	
C4	1.19938 (9)	0.67648 (7)	0.48422 (13)	0.0201 (2)	
C5	1.12617 (9)	0.65686 (6)	0.39571 (13)	0.0178 (2)	
C6	1.07802 (9)	0.70427 (6)	0.31049 (12)	0.0164 (2)	
C7	0.99820 (9)	0.68861 (6)	0.21888 (12)	0.0158 (2)	
C8	0.95183 (9)	0.73961 (6)	0.14831 (12)	0.0151 (2)	
C9	0.98850 (9)	0.80588 (6)	0.16423 (12)	0.0163 (2)	
C10	0.96496 (10)	0.61872 (6)	0.20841 (13)	0.0204 (3)	
C11	1.02484 (14)	0.56977 (8)	0.16651 (16)	0.0306 (3)	
C12	0.99534 (18)	0.50358 (8)	0.16652 (19)	0.0442 (5)	
C13	0.90824 (17)	0.48653 (9)	0.2083 (2)	0.0462 (5)	
C14	0.84867 (14)	0.53492 (8)	0.24773 (19)	0.0397 (5)	
C15	0.87629 (11)	0.60117 (7)	0.24724 (15)	0.0256 (3)	
C16	0.86764 (9)	0.72709 (6)	0.05412 (12)	0.0155 (2)	
C17	0.86503 (10)	0.68158 (7)	−0.06366 (12)	0.0181 (2)	
C18	0.75850 (10)	0.68279 (6)	−0.11310 (12)	0.0172 (2)	
C19	0.70807 (9)	0.62211 (6)	−0.06680 (12)	0.0172 (2)	
C20	0.70797 (9)	0.56215 (6)	−0.14227 (12)	0.0182 (2)	
C21	0.63758 (9)	0.50178 (6)	0.01225 (13)	0.0189 (2)	
C22	0.60415 (10)	0.43954 (7)	0.05349 (15)	0.0223 (3)	
C23	0.56744 (11)	0.43584 (8)	0.17411 (16)	0.0266 (3)	
C24	0.56202 (11)	0.49167 (8)	0.25571 (15)	0.0278 (3)	
C25	0.59295 (11)	0.55210 (8)	0.21628 (14)	0.0241 (3)	
C26	0.63158 (10)	0.55810 (7)	0.09349 (13)	0.0195 (2)	
C27	0.66770 (10)	0.61885 (7)	0.04946 (13)	0.0192 (2)	
C28	0.63772 (9)	0.77074 (6)	−0.06672 (12)	0.0171 (2)	
C29	0.57125 (10)	0.74588 (7)	−0.16539 (14)	0.0222 (3)	
C30	0.48047 (11)	0.77291 (8)	−0.18384 (16)	0.0269 (3)	
C31	0.45373 (11)	0.82413 (8)	−0.10524 (16)	0.0285 (3)	
C32	0.51985 (11)	0.84924 (8)	−0.00833 (14)	0.0265 (3)	
C33	0.61164 (10)	0.82371 (7)	0.01109 (13)	0.0207 (2)	
C34	0.73866 (13)	0.51077 (8)	−0.34404 (16)	0.0292 (3)	
C35	0.60857 (12)	0.38028 (7)	−0.03465 (17)	0.0278 (3)	
C36	0.94255 (10)	0.86304 (7)	0.08870 (14)	0.0204 (2)	
H2	1.2038 (15)	0.8352 (11)	0.418 (2)	0.039 (6)*	
H3	1.2803 (15)	0.7556 (11)	0.559 (2)	0.039 (6)*	
H5	1.1052 (13)	0.6108 (9)	0.3924 (18)	0.023 (4)*	
H11	1.0823 (13)	0.5813 (9)	0.1388 (17)	0.020 (4)*	
H12	1.0391 (18)	0.4749 (13)	0.137 (2)	0.057 (7)*	
H13	0.8908 (16)	0.4411 (12)	0.208 (2)	0.046 (6)*	
H15	0.8328 (13)	0.6360 (9)	0.2763 (18)	0.025 (5)*	
H14	0.7855 (17)	0.5205 (12)	0.275 (2)	0.050 (7)*	
H17A	0.9034 (12)	0.7012 (9)	−0.1273 (17)	0.020 (4)*	
H17B	0.8907 (12)	0.6379 (9)	−0.0441 (17)	0.017 (4)*	
H18	0.7489 (12)	0.6872 (8)	−0.2091 (17)	0.017 (4)*	
H22	0.5425 (14)	0.3943 (10)	0.2025 (19)	0.030 (5)*	
H24	0.5361 (15)	0.4868 (11)	0.339 (2)	0.041 (6)*	
H25	0.5903 (14)	0.5906 (10)	0.271 (2)	0.031 (5)*	
H27	0.6655 (13)	0.6569 (9)	0.1041 (18)	0.026 (5)*	
H29	0.5881 (13)	0.7098 (10)	−0.2214 (19)	0.028 (5)*	
H30	0.4387 (14)	0.7550 (10)	−0.251 (2)	0.030 (5)*	
H31	0.3914 (15)	0.8436 (10)	−0.117 (2)	0.037 (5)*	
H32	0.5040 (14)	0.8860 (10)	0.0468 (19)	0.029 (5)*	
H33	0.6560 (13)	0.8420 (9)	0.0747 (19)	0.027 (5)*	
H34A	0.7653 (15)	0.5244 (11)	−0.424 (2)	0.038 (6)*	
H34B	0.7735 (15)	0.4747 (11)	−0.298 (2)	0.037 (5)*	
H34C	0.6709 (15)	0.4980 (10)	−0.365 (2)	0.033 (5)*	
H35A	0.6724 (16)	0.3750 (11)	−0.067 (2)	0.042 (6)*	
H35B	0.5669 (16)	0.3864 (11)	−0.115 (2)	0.042 (6)*	
H35C	0.5922 (16)	0.3408 (12)	0.011 (2)	0.044 (6)*	
H36A	0.9858 (14)	0.8999 (10)	0.0947 (19)	0.027 (5)*	
H36B	0.9251 (13)	0.8521 (9)	−0.0029 (19)	0.027 (5)*	
H36C	0.8859 (14)	0.8752 (9)	0.127 (2)	0.029 (5)*	

### Atomic displacement parameters (Å^2^)

**Table:**

	*U*^11^	*U*^22^	*U*^33^	*U*^12^	*U*^13^	*U*^23^
Cl1	0.02182 (15)	0.02468 (16)	0.02474 (16)	0.00263 (12)	−0.00384 (11)	0.00766 (12)
O1	0.0323 (5)	0.0186 (5)	0.0175 (4)	−0.0014 (4)	0.0039 (4)	−0.0057 (3)
N1	0.0191 (5)	0.0153 (5)	0.0246 (5)	−0.0015 (4)	−0.0016 (4)	0.0040 (4)
N2	0.0211 (5)	0.0145 (5)	0.0131 (4)	0.0007 (4)	−0.0026 (4)	−0.0008 (3)
N3	0.0226 (5)	0.0156 (5)	0.0156 (5)	0.0036 (4)	−0.0048 (4)	−0.0046 (4)
N4	0.0207 (5)	0.0160 (5)	0.0198 (5)	0.0024 (4)	−0.0038 (4)	−0.0019 (4)
C1	0.0174 (5)	0.0162 (6)	0.0237 (6)	−0.0019 (4)	−0.0025 (5)	0.0035 (4)
C2	0.0256 (7)	0.0195 (7)	0.0417 (9)	−0.0067 (5)	−0.0132 (6)	0.0058 (6)
C3	0.0230 (6)	0.0246 (7)	0.0374 (8)	−0.0052 (5)	−0.0128 (6)	0.0063 (6)
C4	0.0182 (5)	0.0206 (6)	0.0208 (6)	0.0015 (5)	−0.0020 (4)	0.0054 (5)
C5	0.0190 (5)	0.0154 (6)	0.0189 (6)	0.0014 (4)	0.0013 (4)	0.0019 (4)
C6	0.0171 (5)	0.0152 (5)	0.0168 (5)	0.0006 (4)	0.0011 (4)	0.0014 (4)
C7	0.0199 (5)	0.0136 (5)	0.0139 (5)	0.0008 (4)	0.0013 (4)	−0.0005 (4)
C8	0.0183 (5)	0.0141 (5)	0.0129 (5)	0.0008 (4)	0.0007 (4)	−0.0008 (4)
C9	0.0178 (5)	0.0146 (5)	0.0168 (5)	0.0004 (4)	0.0022 (4)	0.0021 (4)
C10	0.0307 (7)	0.0128 (5)	0.0160 (5)	0.0004 (5)	−0.0074 (5)	0.0001 (4)
C11	0.0453 (9)	0.0196 (7)	0.0253 (7)	0.0092 (6)	−0.0056 (6)	−0.0045 (5)
C12	0.0746 (14)	0.0187 (7)	0.0349 (9)	0.0165 (8)	−0.0187 (9)	−0.0067 (6)
C13	0.0659 (13)	0.0164 (7)	0.0494 (11)	−0.0091 (8)	−0.0313 (10)	0.0056 (7)
C14	0.0447 (10)	0.0240 (8)	0.0445 (10)	−0.0143 (7)	−0.0266 (8)	0.0164 (7)
C15	0.0305 (7)	0.0187 (6)	0.0247 (7)	−0.0061 (5)	−0.0127 (5)	0.0068 (5)
C16	0.0213 (5)	0.0126 (5)	0.0123 (5)	0.0000 (4)	−0.0001 (4)	−0.0008 (4)
C17	0.0229 (6)	0.0177 (6)	0.0136 (5)	0.0023 (5)	0.0008 (4)	−0.0032 (4)
C18	0.0249 (6)	0.0142 (5)	0.0121 (5)	0.0022 (4)	−0.0012 (4)	−0.0026 (4)
C19	0.0216 (6)	0.0150 (5)	0.0144 (5)	0.0027 (4)	−0.0025 (4)	−0.0019 (4)
C20	0.0202 (6)	0.0173 (6)	0.0161 (5)	0.0028 (4)	−0.0028 (4)	−0.0025 (4)
C21	0.0186 (5)	0.0179 (6)	0.0189 (6)	0.0027 (4)	−0.0052 (4)	0.0015 (4)
C22	0.0199 (6)	0.0182 (6)	0.0272 (7)	0.0015 (5)	−0.0061 (5)	0.0035 (5)
C23	0.0246 (6)	0.0248 (7)	0.0293 (7)	−0.0007 (5)	−0.0041 (5)	0.0087 (5)
C24	0.0286 (7)	0.0325 (8)	0.0217 (7)	0.0011 (6)	−0.0001 (5)	0.0087 (6)
C25	0.0267 (7)	0.0266 (7)	0.0186 (6)	0.0023 (5)	0.0003 (5)	0.0020 (5)
C26	0.0214 (6)	0.0201 (6)	0.0161 (5)	0.0024 (5)	−0.0033 (4)	0.0012 (4)
C27	0.0245 (6)	0.0176 (6)	0.0147 (5)	0.0021 (5)	−0.0020 (4)	−0.0017 (4)
C28	0.0213 (6)	0.0153 (5)	0.0145 (5)	0.0013 (4)	−0.0001 (4)	0.0025 (4)
C29	0.0249 (6)	0.0197 (6)	0.0206 (6)	0.0001 (5)	−0.0042 (5)	0.0003 (5)
C30	0.0233 (6)	0.0290 (7)	0.0270 (7)	−0.0021 (6)	−0.0048 (5)	0.0039 (6)
C31	0.0225 (6)	0.0356 (8)	0.0276 (7)	0.0060 (6)	0.0038 (5)	0.0084 (6)
C32	0.0296 (7)	0.0306 (8)	0.0199 (6)	0.0098 (6)	0.0055 (5)	0.0021 (5)
C33	0.0271 (6)	0.0208 (6)	0.0142 (5)	0.0040 (5)	0.0013 (5)	0.0008 (4)
C34	0.0405 (9)	0.0231 (7)	0.0245 (7)	−0.0018 (6)	0.0062 (6)	−0.0116 (5)
C35	0.0274 (7)	0.0174 (6)	0.0371 (8)	0.0008 (5)	−0.0049 (6)	0.0006 (6)
C36	0.0222 (6)	0.0151 (6)	0.0233 (6)	0.0003 (5)	−0.0005 (5)	0.0043 (5)

### Geometric parameters (Å, °)

**Table:**

Cl1—C4	1.7394 (13)	C17—H17A	0.968 (18)
O1—C20	1.3626 (16)	C17—H17B	0.969 (17)
O1—C34	1.4407 (17)	C18—C19	1.5161 (18)
N1—C9	1.3163 (17)	C18—H18	0.979 (17)
N1—C1	1.3673 (17)	C19—C27	1.3633 (18)
N2—C16	1.2917 (17)	C19—C20	1.4361 (17)
N2—N3	1.3835 (15)	C21—C26	1.4153 (19)
N3—C28	1.3895 (17)	C21—C22	1.4218 (19)
N3—C18	1.4722 (16)	C22—C23	1.380 (2)
N4—C20	1.3001 (17)	C22—C35	1.503 (2)
N4—C21	1.3753 (18)	C23—C24	1.409 (2)
C1—C2	1.413 (2)	C23—H22	0.97 (2)
C1—C6	1.4144 (18)	C24—C25	1.371 (2)
C2—C3	1.371 (2)	C24—H24	0.96 (2)
C2—H2	0.97 (2)	C25—C26	1.4160 (19)
C3—C4	1.405 (2)	C25—H25	0.96 (2)
C3—H3	0.97 (2)	C26—C27	1.4193 (19)
C4—C5	1.3658 (19)	C27—H27	0.952 (19)
C5—C6	1.4232 (18)	C28—C33	1.4023 (18)
C5—H5	0.977 (18)	C28—C29	1.4030 (19)
C6—C7	1.4301 (18)	C29—C30	1.390 (2)
C7—C8	1.3864 (17)	C29—H29	0.969 (19)
C7—C10	1.4904 (18)	C30—C31	1.384 (2)
C8—C9	1.4407 (17)	C30—H30	0.93 (2)
C8—C16	1.4783 (18)	C31—C32	1.390 (2)
C9—C36	1.5022 (18)	C31—H31	0.96 (2)
C10—C15	1.394 (2)	C32—C33	1.392 (2)
C10—C11	1.395 (2)	C32—H32	0.97 (2)
C11—C12	1.402 (3)	C33—H33	0.934 (19)
C11—H11	0.914 (18)	C34—H34A	0.97 (2)
C12—C13	1.382 (4)	C34—H34B	0.97 (2)
C12—H12	0.92 (3)	C34—H34C	1.00 (2)
C13—C14	1.375 (3)	C35—H35A	0.99 (2)
C13—H13	0.95 (2)	C35—H35B	0.97 (2)
C14—C15	1.396 (2)	C35—H35C	0.96 (2)
C14—H14	1.00 (2)	C36—H36A	0.96 (2)
C15—H15	0.998 (19)	C36—H36B	0.97 (2)
C16—C17	1.5101 (17)	C36—H36C	0.96 (2)
C17—C18	1.5413 (19)		
			
C20—O1—C34	116.13 (11)	C19—C18—H18	111.1 (10)
C9—N1—C1	118.93 (11)	C17—C18—H18	111.5 (10)
C16—N2—N3	108.41 (10)	C27—C19—C20	116.41 (12)
N2—N3—C28	120.39 (10)	C27—C19—C18	123.79 (11)
N2—N3—C18	111.96 (10)	C20—C19—C18	119.61 (11)
C28—N3—C18	125.06 (11)	N4—C20—O1	119.93 (11)
C20—N4—C21	117.81 (11)	N4—C20—C19	125.56 (12)
N1—C1—C2	117.45 (12)	O1—C20—C19	114.51 (11)
N1—C1—C6	122.92 (12)	N4—C21—C26	121.64 (12)
C2—C1—C6	119.62 (12)	N4—C21—C22	118.35 (12)
C3—C2—C1	120.80 (14)	C26—C21—C22	120.01 (12)
C3—C2—H2	120.2 (13)	C23—C22—C21	118.29 (13)
C1—C2—H2	119.0 (13)	C23—C22—C35	121.95 (14)
C2—C3—C4	119.24 (13)	C21—C22—C35	119.75 (13)
C2—C3—H3	120.4 (13)	C22—C23—C24	121.87 (14)
C4—C3—H3	120.4 (13)	C22—C23—H22	119.5 (12)
C5—C4—C3	121.87 (12)	C24—C23—H22	118.6 (12)
C5—C4—Cl1	120.03 (11)	C25—C24—C23	120.36 (14)
C3—C4—Cl1	118.10 (10)	C25—C24—H24	120.6 (13)
C4—C5—C6	119.75 (12)	C23—C24—H24	119.0 (13)
C4—C5—H5	120.6 (11)	C24—C25—C26	119.67 (14)
C6—C5—H5	119.7 (11)	C24—C25—H25	121.4 (12)
C1—C6—C5	118.70 (12)	C26—C25—H25	118.9 (12)
C1—C6—C7	117.75 (11)	C21—C26—C25	119.79 (13)
C5—C6—C7	123.47 (12)	C21—C26—C27	117.97 (12)
C8—C7—C6	118.62 (11)	C25—C26—C27	122.21 (13)
C8—C7—C10	122.57 (11)	C19—C27—C26	120.55 (12)
C6—C7—C10	118.72 (11)	C19—C27—H27	120.6 (11)
C7—C8—C9	119.11 (11)	C26—C27—H27	118.8 (11)
C7—C8—C16	121.43 (11)	N3—C28—C33	121.25 (12)
C9—C8—C16	119.43 (11)	N3—C28—C29	119.68 (12)
N1—C9—C8	122.57 (11)	C33—C28—C29	119.05 (12)
N1—C9—C36	115.77 (11)	C30—C29—C28	120.18 (14)
C8—C9—C36	121.66 (11)	C30—C29—H29	119.3 (11)
C15—C10—C11	119.50 (14)	C28—C29—H29	120.5 (11)
C15—C10—C7	120.43 (12)	C31—C30—C29	120.95 (14)
C11—C10—C7	119.97 (14)	C31—C30—H30	121.8 (12)
C10—C11—C12	119.29 (19)	C29—C30—H30	117.3 (12)
C10—C11—H11	119.6 (11)	C30—C31—C32	118.89 (14)
C12—C11—H11	121.1 (11)	C30—C31—H31	122.2 (13)
C13—C12—C11	120.74 (18)	C32—C31—H31	118.9 (13)
C13—C12—H12	126.1 (16)	C31—C32—C33	121.34 (14)
C11—C12—H12	113.2 (16)	C31—C32—H32	121.0 (12)
C14—C13—C12	119.91 (16)	C33—C32—H32	117.6 (12)
C14—C13—H13	121.5 (14)	C32—C33—C28	119.55 (13)
C12—C13—H13	118.5 (14)	C32—C33—H33	120.4 (12)
C13—C14—C15	120.25 (19)	C28—C33—H33	120.0 (12)
C13—C14—H14	117.3 (14)	O1—C34—H34A	105.0 (13)
C15—C14—H14	122.4 (14)	O1—C34—H34B	108.5 (12)
C10—C15—C14	120.28 (17)	H34A—C34—H34B	113.3 (17)
C10—C15—H15	119.9 (11)	O1—C34—H34C	110.3 (12)
C14—C15—H15	119.9 (11)	H34A—C34—H34C	109.5 (17)
N2—C16—C8	121.23 (11)	H34B—C34—H34C	110.0 (17)
N2—C16—C17	112.85 (11)	C22—C35—H35A	111.8 (13)
C8—C16—C17	125.37 (11)	C22—C35—H35B	110.4 (13)
C16—C17—C18	101.58 (10)	H35A—C35—H35B	103.4 (18)
C16—C17—H17A	108.0 (10)	C22—C35—H35C	110.2 (14)
C18—C17—H17A	111.4 (10)	H35A—C35—H35C	109.7 (18)
C16—C17—H17B	114.3 (10)	H35B—C35—H35C	111.2 (18)
C18—C17—H17B	114.6 (10)	C9—C36—H36A	108.8 (12)
H17A—C17—H17B	106.8 (14)	C9—C36—H36B	112.2 (11)
N3—C18—C19	112.57 (11)	H36A—C36—H36B	109.9 (16)
N3—C18—C17	100.46 (10)	C9—C36—H36C	108.9 (12)
C19—C18—C17	110.97 (10)	H36A—C36—H36C	109.1 (16)
N3—C18—H18	109.8 (10)	H36B—C36—H36C	108.0 (16)
			
C16—N2—N3—C28	175.71 (12)	N2—N3—C18—C19	97.03 (13)
C16—N2—N3—C18	13.06 (14)	C28—N3—C18—C19	−64.65 (16)
C9—N1—C1—C2	−176.86 (14)	N2—N3—C18—C17	−21.06 (13)
C9—N1—C1—C6	2.4 (2)	C28—N3—C18—C17	177.26 (12)
N1—C1—C2—C3	177.85 (16)	C16—C17—C18—N3	19.72 (12)
C6—C1—C2—C3	−1.4 (2)	C16—C17—C18—C19	−99.54 (11)
C1—C2—C3—C4	0.5 (3)	N3—C18—C19—C27	−22.39 (18)
C2—C3—C4—C5	0.3 (2)	C17—C18—C19—C27	89.31 (15)
C2—C3—C4—Cl1	−179.73 (14)	N3—C18—C19—C20	162.75 (11)
C3—C4—C5—C6	−0.3 (2)	C17—C18—C19—C20	−85.55 (14)
Cl1—C4—C5—C6	179.75 (10)	C21—N4—C20—O1	−179.46 (11)
N1—C1—C6—C5	−177.81 (12)	C21—N4—C20—C19	0.3 (2)
C2—C1—C6—C5	1.4 (2)	C34—O1—C20—N4	5.31 (18)
N1—C1—C6—C7	−0.9 (2)	C34—O1—C20—C19	−174.44 (12)
C2—C1—C6—C7	178.31 (13)	C27—C19—C20—N4	−2.2 (2)
C4—C5—C6—C1	−0.57 (19)	C18—C19—C20—N4	172.99 (12)
C4—C5—C6—C7	−177.28 (12)	C27—C19—C20—O1	177.49 (12)
C1—C6—C7—C8	−1.97 (18)	C18—C19—C20—O1	−7.27 (17)
C5—C6—C7—C8	174.77 (12)	C20—N4—C21—C26	1.45 (19)
C1—C6—C7—C10	−178.68 (12)	C20—N4—C21—C22	−178.37 (12)
C5—C6—C7—C10	−1.95 (19)	N4—C21—C22—C23	179.02 (12)
C6—C7—C8—C9	3.31 (17)	C26—C21—C22—C23	−0.81 (19)
C10—C7—C8—C9	179.89 (12)	N4—C21—C22—C35	−1.63 (19)
C6—C7—C8—C16	−178.70 (11)	C26—C21—C22—C35	178.54 (12)
C10—C7—C8—C16	−2.12 (19)	C21—C22—C23—C24	0.3 (2)
C1—N1—C9—C8	−0.94 (19)	C35—C22—C23—C24	−179.01 (14)
C1—N1—C9—C36	178.32 (12)	C22—C23—C24—C25	0.5 (2)
C7—C8—C9—N1	−1.94 (19)	C23—C24—C25—C26	−0.8 (2)
C16—C8—C9—N1	−179.96 (12)	N4—C21—C26—C25	−179.30 (12)
C7—C8—C9—C36	178.85 (12)	C22—C21—C26—C25	0.52 (19)
C16—C8—C9—C36	0.82 (18)	N4—C21—C26—C27	−1.13 (19)
C8—C7—C10—C15	−63.05 (17)	C22—C21—C26—C27	178.69 (12)
C6—C7—C10—C15	113.53 (14)	C24—C25—C26—C21	0.3 (2)
C8—C7—C10—C11	120.55 (15)	C24—C25—C26—C27	−177.82 (14)
C6—C7—C10—C11	−62.87 (17)	C20—C19—C27—C26	2.46 (19)
C15—C10—C11—C12	−1.1 (2)	C18—C19—C27—C26	−172.55 (12)
C7—C10—C11—C12	175.28 (14)	C21—C26—C27—C19	−0.95 (19)
C10—C11—C12—C13	−0.4 (3)	C25—C26—C27—C19	177.18 (13)
C11—C12—C13—C14	1.4 (3)	N2—N3—C28—C33	10.38 (19)
C12—C13—C14—C15	−0.8 (3)	C18—N3—C28—C33	170.62 (12)
C11—C10—C15—C14	1.8 (2)	N2—N3—C28—C29	−171.60 (12)
C7—C10—C15—C14	−174.63 (13)	C18—N3—C28—C29	−11.36 (19)
C13—C14—C15—C10	−0.8 (2)	N3—C28—C29—C30	−179.09 (13)
N3—N2—C16—C8	173.70 (11)	C33—C28—C29—C30	−1.0 (2)
N3—N2—C16—C17	1.81 (15)	C28—C29—C30—C31	−0.5 (2)
C7—C8—C16—N2	131.09 (13)	C29—C30—C31—C32	1.1 (2)
C9—C8—C16—N2	−50.93 (17)	C30—C31—C32—C33	−0.3 (2)
C7—C8—C16—C17	−58.08 (17)	C31—C32—C33—C28	−1.3 (2)
C9—C8—C16—C17	119.90 (14)	N3—C28—C33—C32	179.91 (13)
N2—C16—C17—C18	−14.56 (14)	C29—C28—C33—C32	1.9 (2)
C8—C16—C17—C18	173.95 (11)		

### Hydrogen-bond geometry (Å, °)

**Table:**

*D*—H···*A*	*D*—H	H···*A*	*D*···*A*	*D*—H···*A*
C13—H13···N1^i^	0.95 (2)	2.56 (2)	3.410 (2)	149 (2)
C14—H14···Cg1	1.00 (2)	2.92 (2)	3.685 (2)	134 (2)
C17—H17A···Cg2^ii^	0.97 (2)	2.55 (2)	3.5018 (14)	169 (1)
C25—H25···Cg3^iii^	0.96 (2)	2.79 (2)	3.7359 (17)	169 (2)

Symmetry codes: (i) −*x*+2, *y*−1/2, −*z*+1/2;  (ii) *x*, −*y*+1/2, *z*−3/2; (iii) *x*, −*y*+1/2, *z*−1/2.
